# Comparison of MOLLI and ShMOLLI at 1.5 and 3 Tesla for detection of early cardiac iron deposition in patients with transfusional siderosis

**DOI:** 10.1186/1532-429X-18-S1-P123

**Published:** 2016-01-27

**Authors:** Andrew L Cheng, John Wood

**Affiliations:** Pediatric Cardiology, Children's Hospital Los Angeles, Los Angeles, CA USA

## Background

Patients receiving chronic transfusion develop iron overload in the liver, endocrine glands, and heart. Cardiac T2* has become the standard of care for detecting preclinical cardiac iron deposition and has been validated against tissue iron levels. Recent work suggests that T1 mapping can also be used to detect myocardial iron and may be more sensitive to early cardiac iron deposition. However, myocardial T1 measurements have not been as well standardized across pulse sequences, vendors, and field strengths. We compared two modified Look-Locker sequences (MOLLI and ShMOLLI) at 1.5 and 3 Tesla in patients with transfusional siderosis.

## Methods

17 patients were scanned using a 16-element torso coil in a Philips Achieva 3T scanner; 9 of these were also scanned at 1.5T. MOLLI (3-3-5 sampling) and ShMOLLI (7 inversion times) images were collected from a single mid-papillary slice in a single breath-hold, with inversion times between 161 and 3900 ms. Epicardial and endocardial boundaries were traced on individual images and segmented according to the AHA 17-segment model. Median T1_MOLLI_ and T1_ShMOLLI_ were compared using Bland-Altman analysis.

## Results

**Figure 1:**
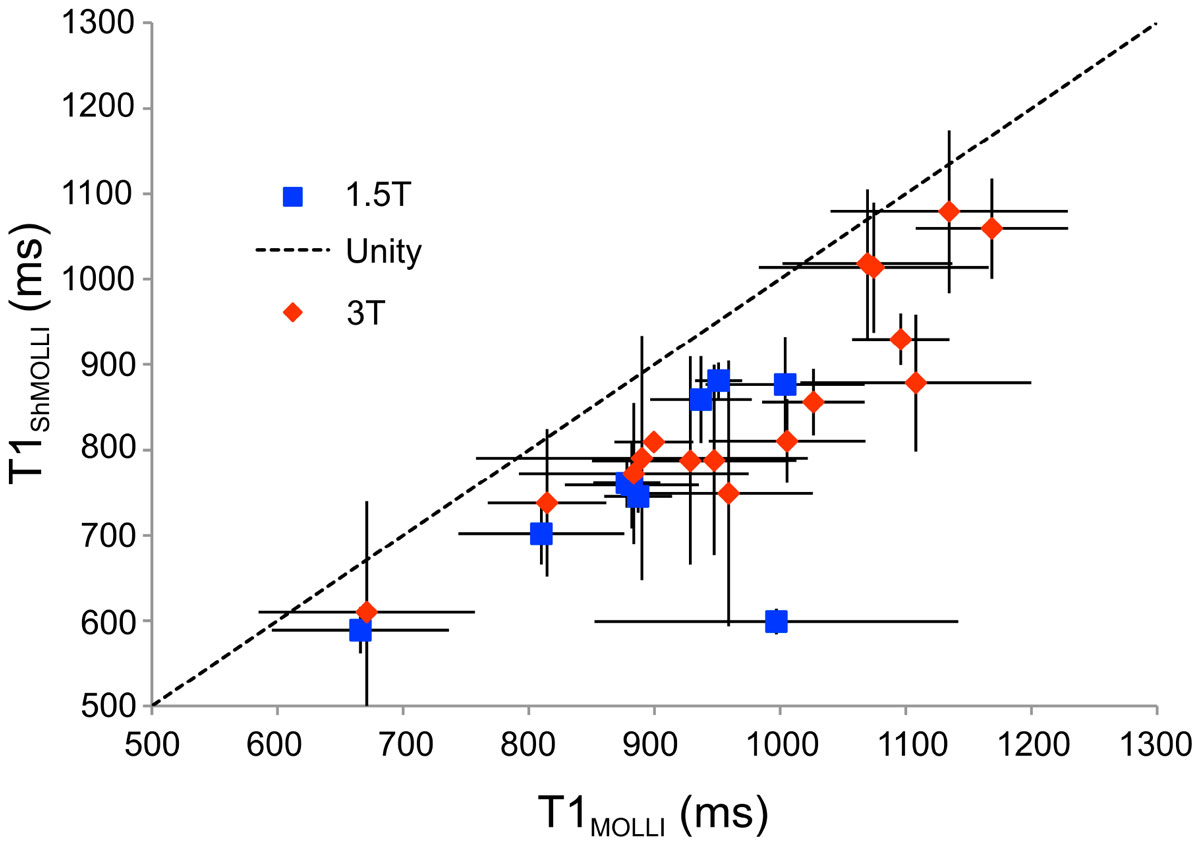
**Comparison of T1 values obtained by MOLLI vs. ShMOLLI**.

**Figure 2:**
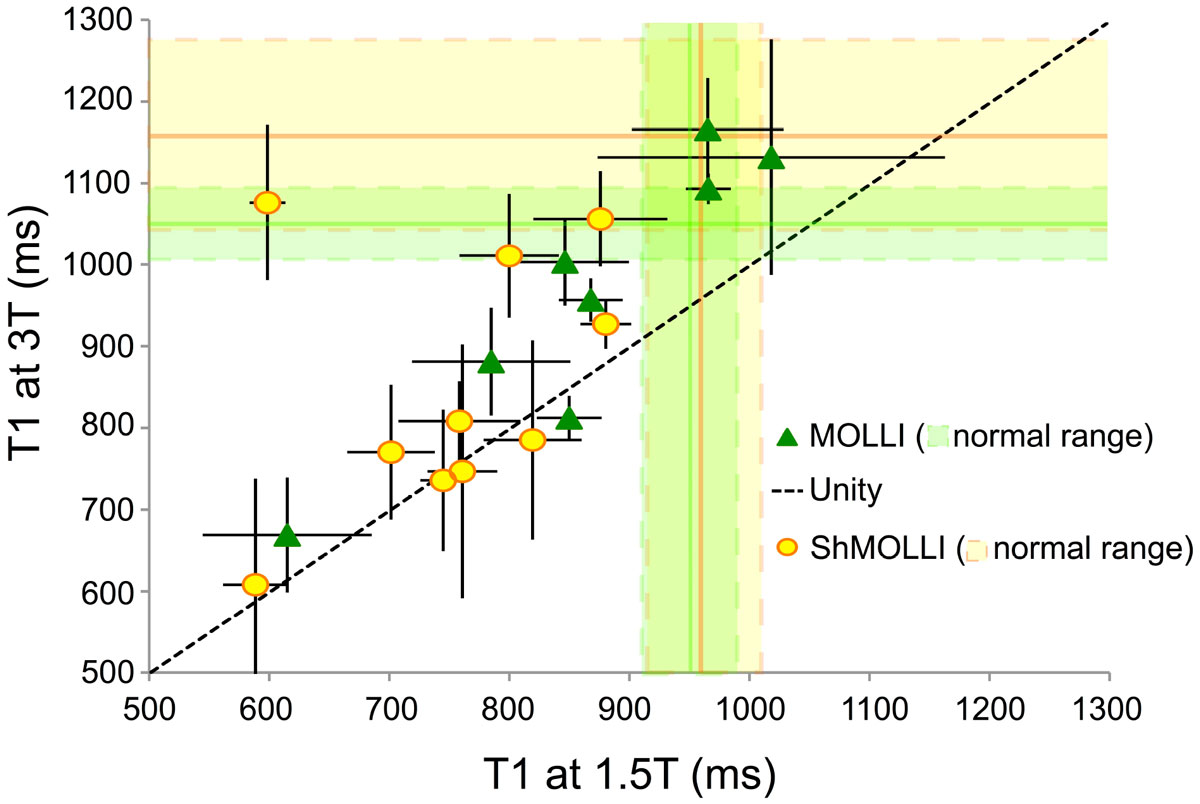
**Comparison of T1 values obtained at 1.5 Tesla vs. 3 Tesla**.

Patients had a mean age of 24.3 ± 11 years, mean BSA of 1.6 ± 0.3 m^2^, and were 53% female. 11 (64.7%) had thalassemia major and the remainder had sickle cell disease. Patients had a broad range of liver iron loading (20.8 ± 11.1, range 0.8-55.8 mg/g), but significant cardiac iron loading (T2* <20 ms) was found in only one subject (range 4.2-45.2 ms). As a result, no significant relationship was seen between cardiac T1 and cardiac T2* by either T1 technique. However, 8 patients had cardiac T2* between 20-30 ms, suggestive of early cardiac iron loading. Also, in the majority of patients, both T1_MOLLI_ and T1_ShMOLLI_ were lower than published norms. Figure 1 demonstrates the relationship between T1_MOLLI_ and T1_ShMOLLI_ at the two field strengths. Line of unity is shown for reference. T1_ShMOLLI_ values were systematically lower than T1_MOLLI_ values. Bland-Altman limits of agreement were -81 ± 26 ms at 1.5T and -124 ± 59 ms at 3T (both p <0.0001). This relationship was independent of field strength. Figure 2 demonstrates T1 values at 3T compared with T1 values at 1.5T for both MOLLI and ShMOLLI. T1 values at 3 Tesla were roughly 25% higher than corresponding values at 1.5 Tesla.

## Conclusions

T2* is gold standard for stratifying cardiac risk in iron overloaded subjects. T1 mapping could complement T2* assessment since it may have better sensitivity and specificity for detecting early cardiac iron deposition, whereas cardiac T2* assessments are more vulnerable to motion and susceptibility artifacts. T1 is also sensitive to fibrosis, and changes with contrast administration could be used to quantify extracellular volume. Unfortunately, there are many T1 mapping techniques (eg. MOLLI, ShMOLLI, SASHA) and they differ in their absolute quantification. In our implementation, ShMOLLI systematically underestimates MOLLI T1 estimates.

